# Circulating exosomal miR-144-3p inhibits the mobilization of endothelial progenitor cells post myocardial infarction via regulating the MMP9 pathway

**DOI:** 10.18632/aging.103651

**Published:** 2020-08-25

**Authors:** Yihai Liu, Jiamin Xu, Rong Gu, Zhu Li, Kun Wang, Yu Qi, Xuan Sun, Jun Xie, Lian Wang, Biao Xu, Lina Kang

**Affiliations:** 1 Department of Cardiology, Nanjing Drum Tower Hospital, Clinical College of Nanjing Medical University, Nanjing 210008, China; 2 Department of Cardiology, Nanjing Drum Tower Hospital, Nanjing University Medical School, Nanjing 210008, Jiangsu, China

**Keywords:** miR-144-3p, endothelial progenitor cells, diabetes, myocardial infarction

## Abstract

Background: The angiogenesis post myocardial infarction (MI) is compromised in diabetes. MiR-144-3p is reported to be highly expressed in circulating exosomes of diabetic patients, implying its role in diabetic complications. However, whether circulating exosomes and enriched miR-144-3p are involved in the impaired neovascularization in diabetes and the underlying mechanism is unclear.

Results: DMexo and miR-144-3p mimic-treated MSCs had elevated miR-144-3p levels and decreased MMP9, Ets1 and PLG expression. The percentage of EPCs were relatively lower in DMexo-treated or agomir-treated MI mice compared with MI mice. Finally, the luciferase assay confirmed the direct binding between miR-144-3p and Ets1.

Conclusion: Exosomal miR-144-3p could impair the mobilization ability of EPCs, which was associated with impaired ischemia-induced neovascularization.

Methods: Circulating exosomes were isolated from Streptozotocin (STZ)-induced mice. *In vitro*, mesenchymal stem cells (MSCs) were incubated with exosomes from diabetic mice (DMexo), and miR-144-3p mimic or inhibitor. miR-144-3p, and MMP9 pathway were measured using qPCR and immunoblotting. *In vivo*, MI mice induced by left anterior descending ligation were treated with DMexo, as well as miR-144-3p agomir. Flow cytometry was used to profile endothelial progenitor cells (EPCs) in peripheral blood and bone marrow post 24 hours respectively.

## INTRODUCTION

Diabetic patients are susceptible to vascular complications such as myocardial infarction, ischemic stroke, lower extremity arterial occlusion, and the poor prognosis after an ischemic event, which is largely related to diminished vascular endothelial repair capacity and angiogenesis disorders [1]. Studies show that angiogenesis and collateral formation of ischemic tissues not only depend on the budding and remodeling of in situ blood vessels, but bone marrow-derived endothelial progenitor cells (EPCs) also play an important role in reactive angiogenesis after ischemia [2]. Ander et al. [3] found that EPCs could obviously mobilize from the bone marrow to peripheral circulation after acute myocardial infarction. Afterwards, circulating EPCs were homing into the ischemic heart [4]. Finally, EPCs differentiate into mature endothelial cells, and the repair process of neovascularization begins [5]. However, the number of EPCs is reduced and the function is defective in the blood circulation of diabetic patients and diabetic rats [6].

EPCs exists in the bone marrow (BM) stromal environment, and Matrix metalloproteinase-9 (MMP9)-mediated soluble Kit-ligand (sKitL) release promotes its mobilization into the circulation [7]. Schafer, K. [8] found that Leptin promotes EPC mobilization mediated by activation of MMP-9. Conversely, Impaired MMP-9 activity in bone marrow likely contribute to reduced EPC mobilization in the early postinfarction phase [9]. MMP9 deficiency mice exhibit reduced EPCs mobilization, as well as impaired wound neovascularization and healing after diabetes [10, 11].

Circulating Myocardial microRNAs are highly expressed in patients with MI. Circulating myo-miRs are carried by exosomes and mediate the interaction between ischemic myocardium and BM. Exosomes miRs are transported into BM mononuclear cells (MNCs), and increase the number of circulating EPCs involved in myocardial repair by inhibiting CXCR4 expression [12]. High-throughput sequencing has suggested that miR-144 is highly expressed in circulation exosomes of diabetic patients, suggesting that it may be involved in systemic complications of diabetes. However, whether miR-144 could also participate in the mobilization of BM EPCs and the specific mechanism is still unknown.

## RESULTS

### MiRNA-144-3p was markedly elevated in the plasma exosome from STZ-induced diabetic mice

**Figure 1:**
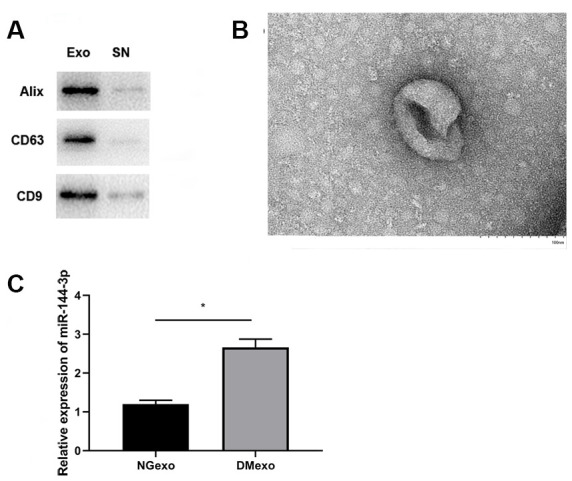
The expression of exosome marker (Alix, CD63, and CD9) in the exosome and supernatant fraction (**A**); the morphology of the diabetic exosome in TEM (**B**), scale bar 100 nm; the relative expression of miR-144-3p between NG-exo and DM-exo groups (**C**). N=3 for each group.

The exosome was rapidly isolated from plasma of diabetic mice using a commercial exosome isolation kit. The pellet (Exo) and the resultant supernatant (SN) were immunoblotted for known exosome marker (Figure 1A). This showed exosome markers, Alix, CD63 and CD9, were expressed in the isolated fraction while not in supernatant fraction. Besides, transmission electron microscopy showed that exosome had a diameter of ~100 nm (Figure 1B), which was in accordance with the reported size. Based on previous studies which profiled circulating microRNAs of type 1 and 2 diabetes [13, 14], we focused on the most dysregulated miR-144-3p. Similarly, compared with the circulating exosome from normal mice (NGexo), qPCR (n=3 each group) confirmed miR-144-3p was upregulated in diabetic exosomes (DMexo; Figure 1C).

### Exosome mediates transfer of circulating miR-144-3p into BM-MSC

**Figure 2:**
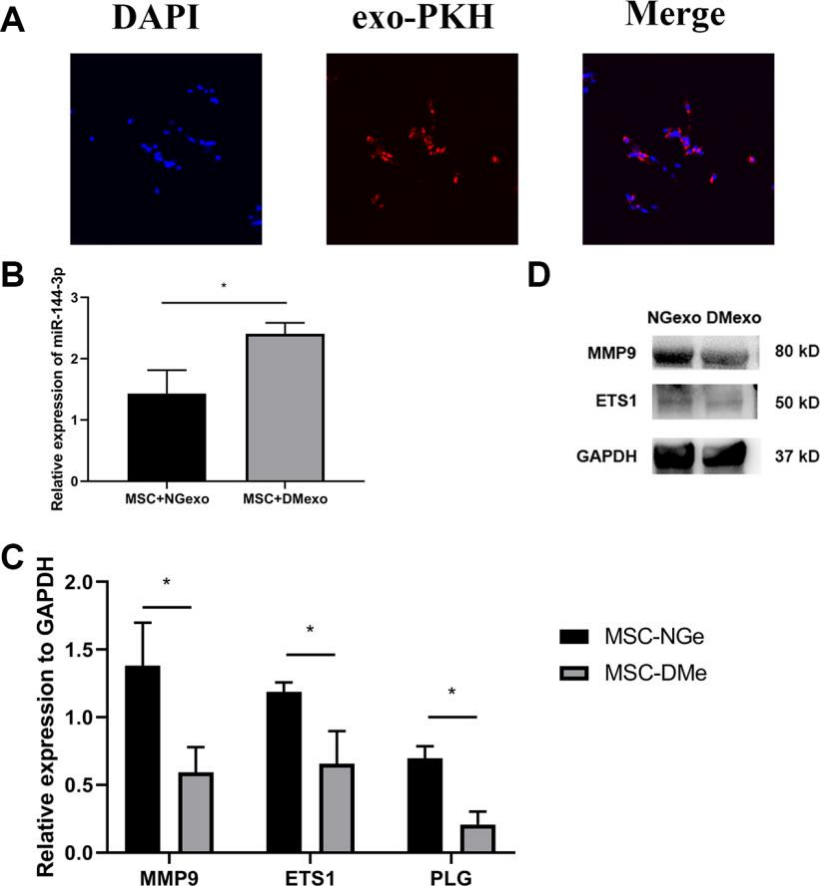
The uptake of diabetic exosomes (red) by MSC (blue) (**A**); Compared with NGexo-treated MSCs, DMexo-treated MSCs showed higher expression levels of miRNA-144-3p (**B**); lower mRNA expression of Mmp9, Ets1 and Plg (**C**); lower protein expression of Mmp9 and Ets1 (**D**).

To investigated whether the exosomes could be transferred to the BM-MSC cells, we labeled exosome with a fluorescent membrane marker PKH67. After coculturing exosome with MSC for 12 hours, the labelled exosomes were taken up by BM-MSCs (Figure 2A). Further, the total RNA and protein of MSC treated with NGexo (20 μg/ml) or DMexo (20 μg/ml) respectively were isolated for subsequent analysis. MSC treated with DMexo had a higher level of miR-144-3p than NGexo-treated MSC (Figure 2B). What’s more, Mmp9, together with Ets1 (regulating expression of Mmp9) and Plg (regulating activity of Mmp9) also showed a decreased expression, either in mRNA (Figure 2C) or protein (Figure 2D).

### MiR-144-3p downregulates MMP9 expression in BM-MSCs *in vitro*

**Figure 3:**
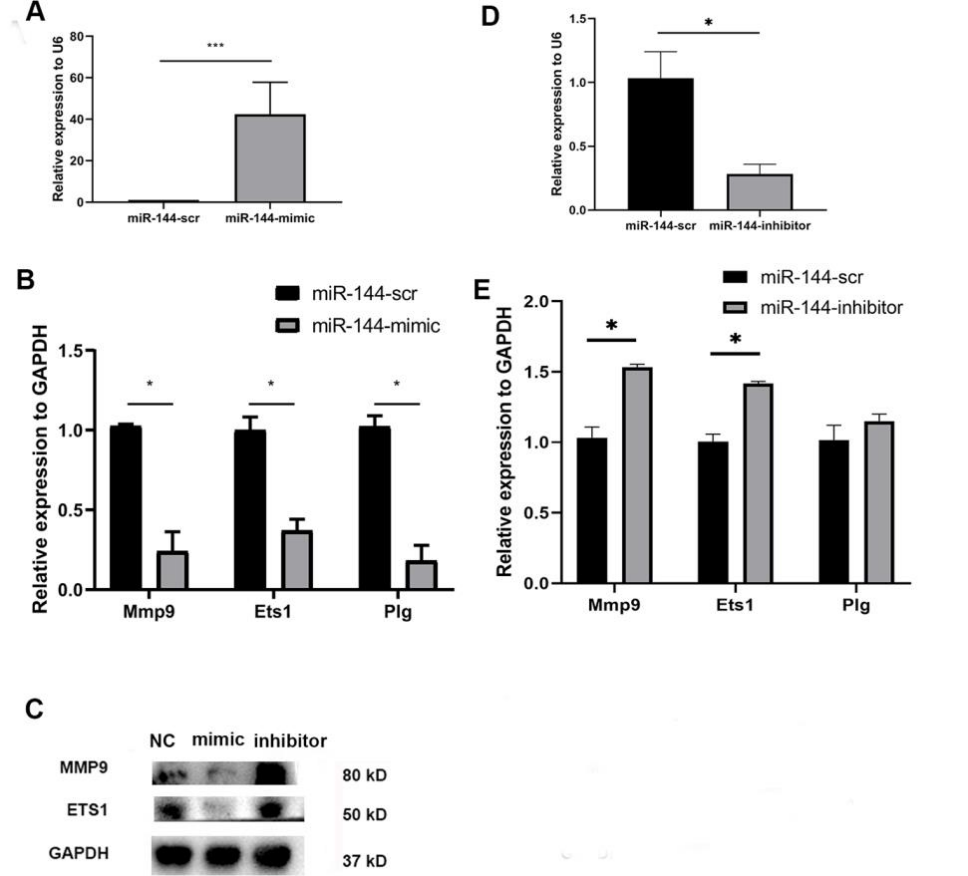
Compared with scramble transfected MSCs, miR-144-3p mimic treated MSCs showed a higher expression of miR-144-3p (**A**); a lower mRNA expression of Mmp9, Ets1 and Plg (**B**); a lower protein expression of MMP9 and Ets1 (**C**). While miR-144-3p inhibitor treated MSCs showed a converse trend (**C**–**E**).

To observe the direct effect of miR-144-3p on MSCs, we transfected MSCs with a synthesized miR-144-3p mimic (50 nM). After 24 hours, the expression of miR-144-3p was significantly upregulated in mimic-treated MSCs (Figure 3A). Besides, mimic group has a lower expression of Mmp9, Ets1 and Plg (Figure 3B). Immunoblotting results also showed that MMP9 and Ets1 were downregulated in MSCs with overexpression of miR-144-3p (Figure 3C). Conversely, miR-144-3p inhibitor could decrease the expression of miR-144-3p (Figure 3D) while upregulated Mmp9, Ets1 and Plg (Figure 3C, 3E) at the mRNA and protein level.

### Ets1 is a direct target of miR-144-3p

**Figure 4:**
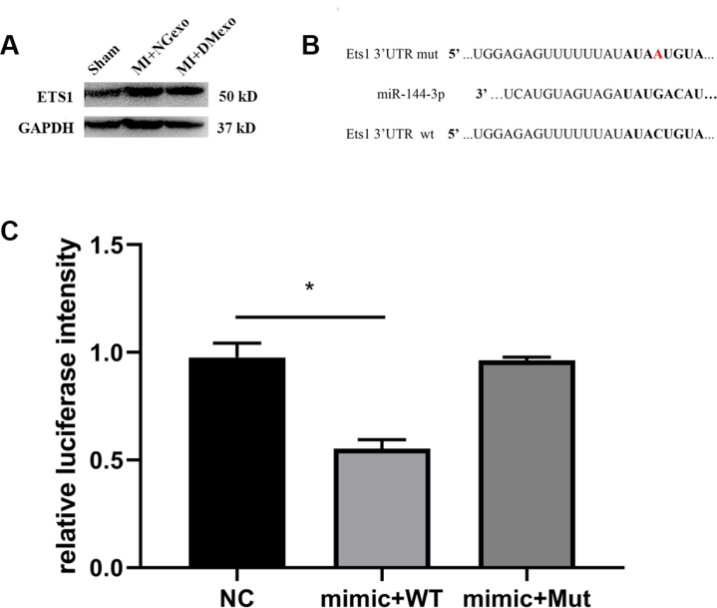
The BM-MSC from MI mice treated with DMexo has a lower Ets1 protein expression compared with NGexo treated MI mice (**A**); the bioinformatic predicted combining sequence of miR-144-3p with 3’-UTR of Ets1 and the mutated 3’-UTR of Ets1 (**B**); the relative luciferase intensity of 293T cells transfected with miR-144-3p mimic in the presence of 3’-UTR of Ets1 or the mutated 3’-UTR (**C**).

Above *in vitro* results showed that the expression of Ets1 was negatively correlated with that of miR-144-3p. Further, MI mice were intravenous administrated into NGexo or DMexo to explore the *in vivo* effect of diabetic exosome. After 12 hours, the hearts were harvested for immunoblotting. We found that Ets1 was upregulated in infarcted heart while abolished by DMexo injection (Figure 4A), confirming the inverse relationship between Ets1 and miR-144-3p. Bioinformatic analysis (http://www.mirdb.org.) predicted that miR-144-3p could bind to 3’UTR of Ets1. So, we constructed a plasmid containing wild type 3’ UTR or mutated 3’ UTR of Ets1 for luciferase measurement (Figure 4B). The reporter results showed that miR-144-3p could target Ets1 and the combining could disappear transfected with the mutated Ets1 (Figure 4C), suggesting that miR-144-3p could directly and specifically bind to Ets1.

### Diabetic exosome inhibits mobilization of BM endothelial progenitor cells post MI

**Figure 5:**
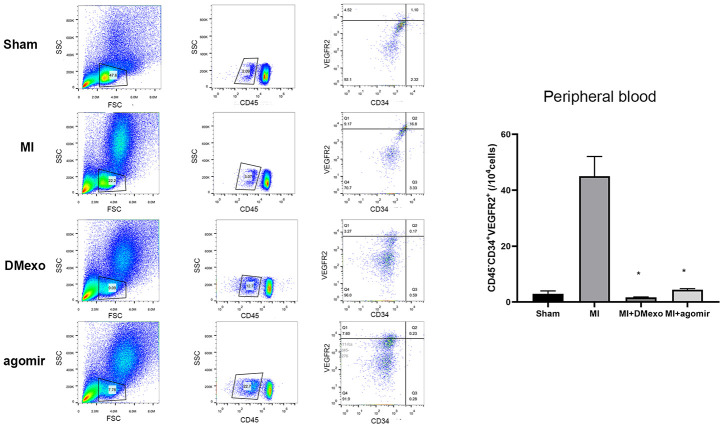
**The percentage of CD45^-^CD34^+^VEGFR2^+^EPCs in the peripheral blood 24 hours post MI among groups.** The quantitative results were shown in the right. *p<0.05 for MI groups.

**Figure 6:**
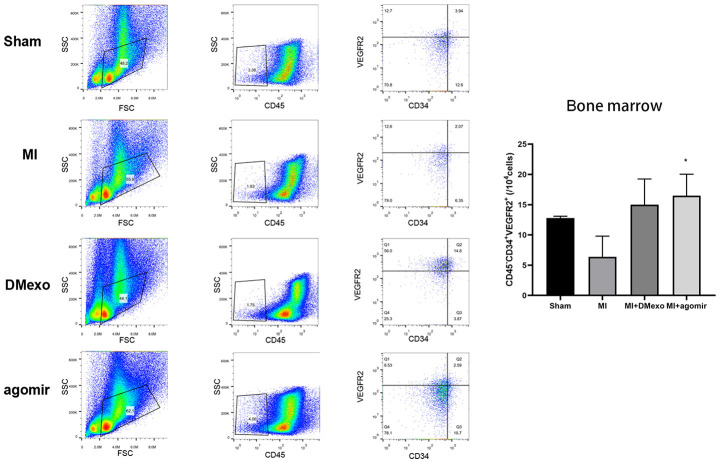
**The percentage of CD45^-^CD34^+^VEGFR2^+^EPCs in the bone marrow 24 hours post MI among groups.** The quantitative results were shown in the right. *p<0.05 for MI groups.

Finally, we investigated whether diabetic exosomes contributed to the impaired mobilization of EPCs post MI. The plasma was harvested from Sham, MI, DMexo and agomir groups. Notably, 24 hours after MI, the PB EPCs were increased in MI group while decreased in DMexo and agomir group (Figure 5), suggesting that DMexo and miR-144-3p could decrease the number of circulating EPCs. While in BM, EPCs were increased in DMexo and agomir group (Figure 6), which could be due to the impaired migration of EPCs in diabetic bone marrow microenvironment.

## DISCUSSION

**Figure 7:**
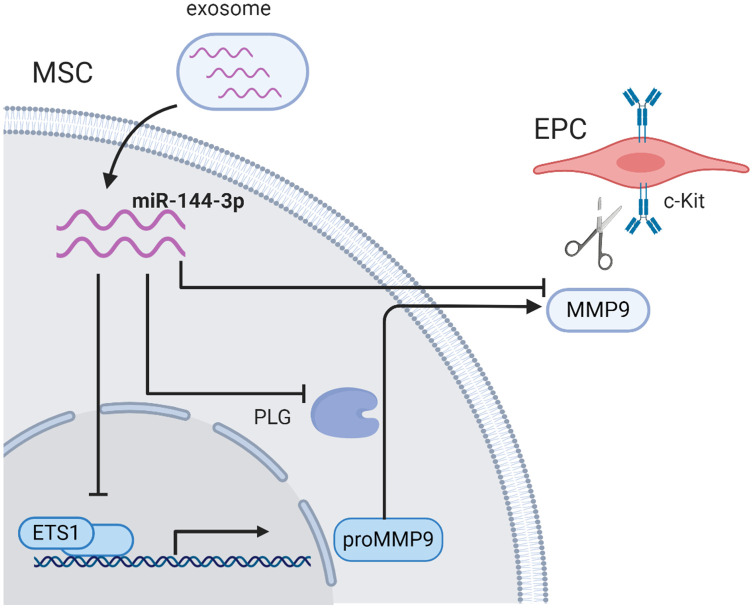
**The schematic illustration of proposed mechanism.** Exosomal miR-144-3p disturbed the MMP-9 pathway by inhibiting Ets1 expression in MSCs, and subsequently impaired the mobilization of EPCs from bone marrow microenvironment.

In the current study, we observed that miR-144-3p was enriched in diabetic exosomes. Upregulated miR-144-3p disturbed the MMP-9 pathway by inhibiting Ets1 expression in MSCs, and subsequently impaired the mobilization of EPCs from bone marrow, which provided a novel mechanism underlying diabetes-mediated re-endothelialization dysfunction post myocardial infarction (Figure 7).

Exosome was suggested to provide beneficial paracrine effects of stem cells on cardiac injury [15, 16]. However, its cardioprotection was impaired in the setting of diabetes [17]. Similarly, we found circulating EPCs were decreased in DMexo-treated MI mice, explaining diabetes-induced angiogenesis dysfunction. In accordance with the previous publication [13], we confirmed that miR-144-3p was enriched in circulation exosomes of diabetes mellitus, suggesting that it may be involved in the complication caused by diabetes. After ischemia, MMP9 promoted neovascularization by modulating bone marrow-derived EPCs [11]. Wound healing function was impaired in diabetic MMP9-/- mice [10], suggesting that MMP9 pathway was necessary for the mobilization of EPCs to circulation. Previous studies reported that MMP9 was downregulated by miR-144-3p in an indirect manner of EZH2-mediated epigenetic regulation [18] or t-PA-mediated activation [19]. Ets1, as a transcription factor of MMP9 [20], was required for endothelial cell survival during angiogenesis [21]. Similarly, our results also showed that decreased Ets1 expression was involved in the dysfunction of EPCs. Combining bioinformatic prediction and luciferase assay, we demonstrated that miR-144-3p inhibited MMP9 expression by silencing Ets1.

The data provided a novel mechanism for diabetes-induced re-endothelialization dysfunction post MI. However, some limitations exist. First, the cargo in exosome was complex and included some complement and inflammatory proteins [22], which could influence the survival and tube forming function of EPCs. Besides, miR-144-3p was reported to targeting the Nrf2 signaling [23] and may enhanced oxidative stress injury in bone marrow environment, causing the exhaustion of progenitor cells. Finally, although EPC mobilization was impaired in DMexo-treated MI mice, it was undetermined whether cardiac function and cardiac remodeling was improved post MI.

In summary, our results found that elevation of miR-144-3p levels in diabetic exosomes could impaired the mobilization ability of EPCs, which was related to compromised ischemia-induced neovascularization. Intervening in the enriched miR-144-3p could be a novel strategy to improve the cardiac repair post myocardial infarction.

## MATERIALS AND METHODS

### Animals and treatments

Male C57BL/6 mice (8w, 20-25g), purchased from Qing Long Shan Animal Breeding Farm (Nanjing, China), were kept in controlled conditions and received a standard mouse chow and tap water ad libitum. The animal work was completed in the central laboratory of Nanjing Drum Tower Hospital. All procedures were in compliance with the Guide for Card and Use of Laboratory Animals and approved by the Animal Care and Use Committee of Nanjing Drum Tower Hospital.

Streptozotocin (STZ; Sigma, USA; 60 mg/kg/d x 5d, i.p.) dissolved in 0.1 mM sodium citrate buffer (pH 4.5) were injected into mice to induce diabetes. After 3 weeks, the mice with the random blood glucose value ≥300 mg/dl were defined as STZ-induced diabetic mice for subsequent plasma exosome extraction.

The myocardial infarction (MI) was induced by permanent ligation of the left anterior descending coronary artery (LAD) as described [24]. The sham groups went through all procedures except for LAD ligation. All mice were anesthetized with 2% isoflurane via face mask. Each group included 3 mice. For miR-144-3p overexpression, mice were treated with miR-144-3p agomir (RiboBio, China; 10 nmol per mouse) by tail vein injection prior to MI surgery. The sequence of miR-144-3p agomir is UACAGUAUAGAUGAUGUACU (5’-3’).

### Isolation of circulating exosomes

Exosomes were isolated from mouse by following protocols. First, the cell debris and platelets were removed by centrifuged at 3000 x g for 15 min from diabetic and normal mouse plasma. Then, 63 μL ExoQuick Exosome Precipitation Solution (SBI, USA) was mixed with each 250 ul platelet-free plasma. The mixture was centrifuged at 1500 g for 30 min at 4 °C to sediment exosomes (20 μg exosomes/250 μL platelet-free plasma). The isolated pellets were fixed in 2% paraformaldehyde and negatively stained with uranyl oxalate for transmission electron microscopy (Servicebio, China). To facilitate tracking *in vitro*, exosomes were labeled with PKH67 Fluorescent Cell Linker Kit (Sigma, USA) according to the manufacturer’s protocol. The amounts of exosomes chosen for use were 50 μg for each mouse and 20 μg for *in vitro* experiments [12, 25].

### Flow cytometry

About 0.2 ml blood was harvested from mice treated with or without diabetic mice-derived exosome (DMexo). The samples were kept in heparin-pretreated tube and labeled with APC-CD45, FITC-CD34, and PE-VEGFR2 (eBioscience, USA) antibodies. Circulating endothelial progenitor cells (EPCs) were defined as CD45^-^CD34^+^VEGFR2^+^ cells. Flowjo (Trees Tar, USA) was used to measure the percentage of EPCs and plot the Figures.

### Mesenchymal stem cell (MSC) culture

Bone marrow derived MSC were obtained from ATCC (USA) and cultured in DMEM supplement with 10% exosome-depleted FBS (Gibco, USA). At 60%-70% confluence, the cells were treated with DMexo or transfected with miR-144-3p mimic (50 nM) or miR-144-3p inhibitor (100 nM) using lipo2000 (RiboBio, China) following the manufacturer’s instructions. For miR-144-3p mimic, the sequence is UACAGUAUAGAUGAUGUACU (5’-3’) while UUUGUACUACACAAAAGUACUG (5’-3’) for negative control. For miR-144-3p inhibitor, the sequence is AGUACAUCAUCUAUACUGUA (5’-3’) while CAGUACUUUUGUGUAGUACAAA for negative control.

### Quantitative RT-PCR analysis

Total RNA was extracted from MSC cells using TRIzol reagent (Takara, China). To extract microRNAs from exosomes, 20 μg exosomes was suspended in 1 ml of TRIzol (Takara, China). After quantified by NanoDrop 2000 (ThermorFisher, USA), 1μg of mRNA and microRNAs were synthesized as cDNA by using HiScript II Q RT SuperMix and miRNA 1^st^ Strand cDNA Synthesis Kit respectively (Vazyme, China). PCR reactions were performed using ChamQ SYBR qPCR Master Mix for mRNA and miRNA Universal SYBR qPCR Master Mix for miRNA (Vazyme, China) in an ABI PRISM 7900 Sequence Detector system (Applied Biosystems, USA). The cycling program was set: 95°C for 5 min; 40 cycles of 95°C for 15 s, 60°C for 30 s and 72°C for 15 s; 60°C for 15 s. Each gene expression was normalized to GAPDH for mRNAs and U6 snRNA for microRNAs. n=3 for each group. The primers are listed as follows: miR-144-3p:F: GCGCGCGTACAGTATAGATGA, R: AGTGCAGGGTCCGAGGTATT; U6 snRNA: F: CTCGCTTCGGCAGCACATATACT, R: ACGCTTCACGAATTTGCGTGTC;GAPDH: F: AGGTCGGTGTGAACGGATTTG, R: GGGGTCGTTGATGGCAACA; MMP9: F: CTGGACAGCCAGACACTAAAG, R: CTCGCGGCAAGTCTTCAGAG; Ets1:F: GATCTCAAGCCGACTCTCACC, R: GACGTGGGTTTCTGTCCACT; PLG: F: GGAGGTGTCTCGGACTGTTTG, R: GATGCCGGTCTTACATTCTGAC.

### Luciferase activity measurement

The wild-type luciferase reporter was constructed with the 3’-UTR of Ets1 containing miR-144-3p binding sites while the mutant type had no miR-144-3p binding sites (GeneCopoeia, Rockville). The wild-type reporter or mutant-type reporter and miR-144-3p mimic or the negative controls were transfected into HEK293 cells using Lipo 2000 (Life Technologies, USA). After 24 hours, the cell medium was collected for luciferase activity measurement. The sequence for 3’-UTR of Ets1 is UGGAGAGUUUUUUAUAUACUGUA while mutated is UGGAGAGUUUUUUAUAUAAUGUA.

### Western blotting

Proteins were extracted using RIPA Lysis buffer (Thermo, USA) with protease inhibitor (Roche, USA). Protein concentration was measured using the BCA kit (Beyotime, China). Then protein was separated by SDS-PAGE and transferred onto PVDF (Millipore, Germany). The membranes were incubated with primary antibodies such as rabbit anti-GAPDH (Abcam; USA), anti-MMP9 (Abcam; USA) and Ets1 (Abcam; USA). The membrane was then incubated with HRP-conjugated secondary antibodies. Finally, proteins were visualized by enhanced chemiluminescence (Amersham, UK).

### Statistic analysis

Data were presented as mean± standard deviation. The difference between groups were analyzed using either Student’s t-test or ANOVA as appropriate. Significance was set at p <0.05.
